# Comparison of Different Visual Feedback Methods for SSVEP-Based BCIs

**DOI:** 10.3390/brainsci10040240

**Published:** 2020-04-18

**Authors:** Mihaly Benda, Ivan Volosyak

**Affiliations:** Faculty of Technology and Bionics, Rhine-Waal University of Applied Sciences, 47533 Kleve, Germany; mihaly.benda@hochschule-rhein-waal.de

**Keywords:** brain–computer interface (BCI), steady-state visual evoked potential (SSVEP), user feedback, electroencephalography (EEG)

## Abstract

In this paper we compared different visual feedback methods, informing users about classification progress in a steady-state visual evoked potential (SSVEP)-based brain–computer interface (BCI) speller application. According to results from our previous studies, changes in stimulus size and contrast as online feedback of classification progress have great impact on BCI performance in SSVEP-based spellers. In this experiment we further investigated these effects, and tested a 4-target SSVEP speller interface with a much higher number of subjects. Five different scenarios were used with variations in stimulus size and contrast, “*no feedback*”, “*size increasing*”, “*size decreasing*”, “*contrast increasing*”, and “*contrast decreasing*”. With each of the five scenarios, 24 participants had to spell six letter words (at least 18 selections with this three-steps speller). The fastest feedback modalities were different for the users, there was no visual feedback which was generally better than the others. With the used interface, six users achieved significantly better Information Transfer Rates (ITRs) compared to the “*no feedback*” condition. Their average improvement by using the individually fastest feedback method was 46.52%. This finding is very important for BCI experiments, as by determining the optimal feedback for the user, the speed of the BCI can be improved without impairing the accuracy.

## 1. Introduction

Brain–computer interfaces (BCIs) use brain signals to control computer applications or external devices. By measuring the normal electrical brain response, i.e., to specific stimuli, an application can be controlled without the need for hand-movements [1]. Electrical brain activity can be measured, for example, by electroencephalography (EEG), which is non-invasive and relatively cheap. These facts, combined with the simple setup using an EEG cap allows BCI experiments with larger subject groups; because the procedure does not involve surgery, just simple and easy setup, the used consumables are cheap to replace, and the price of the equipment allows even using multiple recording stations.

One of the commonly used methods in BCIs are steady-state visual evoked potentials (SSVEPs) which can be detected from the occipital area of the brain when the users are looking at a light source flickering with a constant frequency between 4 and 90 Hz [2]. Flickering light sources are easily realisable, for example, on a computer monitor. The constant frequency of the visual stimulation can then be set based on the vertical refresh rate of the monitor, which further improves the quality of the visual stimuli [3]. There have been tests with a wide variety of flickering objects, from checkerboard stimuli, through simple squares, to human faces [4,5]. Differently coloured stimuli were also tested on multiple occasions [6,7,8].

While most other BCI modalities require training before the first use of the system, SSVEP-based BCIs can be operated with little to no training [9], although training may improve performance. On the other hand, constant flickering can cause visual fatigue or discomfort for some users.

Commonly, BCIs are developed for people with motor disabilities, who have restricted means for communication. While BCIs are a viable solution for communication without muscle use, their speed is still lower as compared to other methods of communication. Therefore, BCIs can benefit greatly from improvements in performance. Several solutions exist targeting BCI performance, such as changes in classification algorithms, or personalising the parameters of the system. In some cases, these personalised parameters result in a faster or an overall more accurate system, for example, bigger stimuli can result in stronger brain responses [10].

Several other BCI parameters can also be adjusted, for example, the flicker frequencies of the stimuli, which manifest in faster or slower flickering. On the one hand, faster flickering can be more user friendly, on the other hand, the brain response is weaker, which can result in inferior performance [11,12]. Another adjustable parameter is the time the flickering stops after selections, usually called gaze shifting time. This defines how much time users have to find the next target, usually the next letter to be spelled via BCI. Longer times make the system slower; however, too short times may result in erroneous classifications. The classification threshold can also be adjusted, as this has an effect on the accuracy and the speed of the BCI; this parameter benefits the most from personalisation [13].

Some stimulus parameters that can influence the amplitude of the evoked neural response are the size [14], contrast [15], and colour [6,7,8] of the stimuli, as well as their distance from one another [16]. Previously, we examined the use of size, contrast, and duty-cycle changes as methods for feedback, as well as a combination of them. According to our results, size and contrast changes resulted in performance increase [17].

As it can be seen from this multitude of parameters, BCIs require, or at least function better with, personalisation. Apart from classification parameters of the BCI, the users themselves influence the performance greatly. This can be the consequence of fatigue, lack of attention, or even the attitude of the user, not to mention muscular activity which can cause noise in the recorded EEG data. Therefore, it seems reasonable to involve the users more in the use of the BCI. In our previous experiments [10,17], we attempted to improve BCI performance by changing these parameters according to the classification results in an online SSVEP-based BCI speller. We found that informing the users about the progress of the target selection can greatly improve performance. We investigated several different feedback methods and found that spelling time can be reduced to 12%–77% of the “*no feedback*” condition using the personalised feedback. The number of participants in these studies was low, however, already with this lower number of subjects we noted only minor effects due to duty-cycle changes. For this reason, and to limit the overall duration of the experiment, in the current study we focused on size and contrast changes.

Although using auditory feedback and not visual, [18] found that their protocol using pitched sound as real-time feedback outperformed classic SSVEP BCIs. In [19], the authors provided real-time feedback for users by changing the colour of the characters in the stimuli every 0.04 s. Tested together with their new classification method based on canonical correlation analysis, they achieved increased performance metrics.

The user comfort was also taken into account during the design phase of the experiment. For some of these stimulus parameters, a better brain response is associated with a lower user comfort [4,15]. To address this issue, we asked the users to rate the different methods on a scale of one to ten, and compared their subjective opinion with the performance results.

In this experiment, we further investigated the effects encountered in our previous studies, by testing an interface with less stimuli (4-target Kleve speller [20,21,22]). We investigated the practical use cases of the feedback mechanisms, therefore, some parameters of the stimuli, such as their size, or the distance between them were set to result in an overall practical BCI system. These settings were already successfully evaluated in many previous studies. We also aimed to get more reliable results by involving more participants in this online BCI experiment.

## 2. Materials and Methods

### 2.1. Subjects

24 subjects (11 female), with a mean (SD) age of 25.2 (4.1) years participated in this study. All participants were healthy adult volunteers. They all gave written informed consent prior to the study in accordance with the Declaration of Helsinki. Information needed for the analysis was stored anonymously; results cannot be traced back to the participants. This study was approved by the Ethical Committee of the Medical Faculty of the University Duisburg-Essen, the ethical approval code is 17-7693-BO. Participants had the opportunity to opt-out of the study at any time. Subjects received a financial reward for their participation in this study.

### 2.2. Hardware

**Figure 1:**
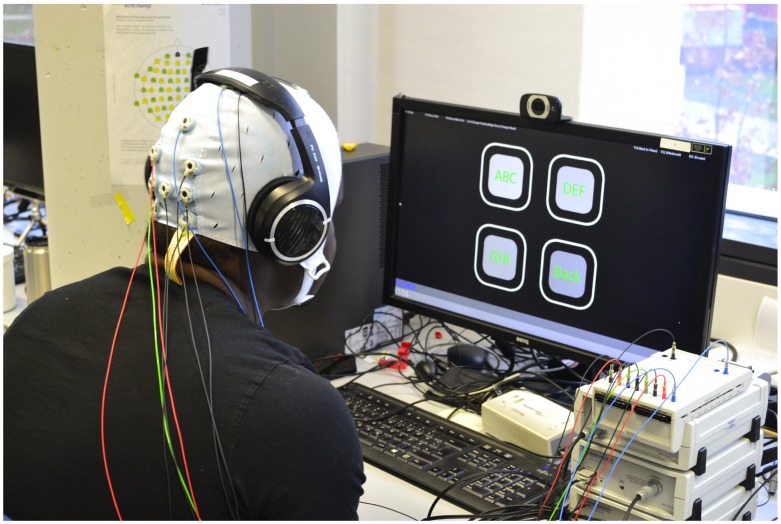
The setup of the experiment with the used 4-target interface. The amplifier, BrainAmp, is located to the right of the monitor.

Participants were seated in front of a LCD screen (BenQ XL2420T, resolution: 1920 × 1080 pixels, vertical refresh rate: 120 Hz) at a distance of about 60 cm, the setup is shown in Figure 1. The used computer system operated on Microsoft Windows 7 Enterprise running on an Intel processor (Intel Core i7, 3.40 GHz). Eight standard Ag/AgCl EEG recording electrodes were used in addition to a ground and a reference electrode to acquire the signals. The ground electrode was placed over $AF_{Z}$, the reference electrode over $C_{Z}$, and the eight signal electrodes were placed at $P_{Z}$, $PO_{3}$, $PO_{4}$, $O_{1}$, $O_{2}$, $O_{Z}$, $O_{9}$, and $O_{10}$ in accordance with the international 10/10 system of EEG electrode placement. Standard abrasive electrode gel was applied between the electrodes and the scalp to bring impedances below 5 kΩ. An EEG amplifier, BrainAmp (Brain Products GmbH, Gilching, Germany), was utilized. The sampling frequency was 5000 Hz, but for classification, the collected EEG data were down-sampled in real time to 250 Hz. A digital band pass filter (between 4 and 40 Hz) was applied on the down-sampled data before classification.

#### 2.2.1. Interface

**Figure 2:**
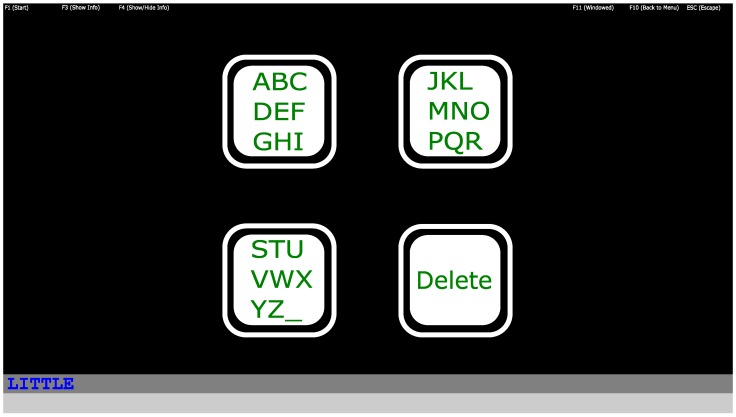
The 4-target interface. The word “LITTLE” notes the target word to be spelled by the subject. The text written by the users was shown at the bottom of the screen, in the line below the target word.

The Graphical User Interface (GUI) of the 4-target interface is shown in Figure 2. For this GUI implementation, each letter required three selections. We have found 4-target systems very robust and controllable by nearly everyone [13]. However, at the same time its drawback is the limited speed, as for each character with this 4-target speller three selections are necessary.

The speller logic was implemented as follows: when a box was firstly selected, its content was spread over three boxes and resulted in three characters per box. After the next selection phase, the three boxes contained only one letter each. With the third selection the selected letter or special character was selected. In the second and the third phases, the fourth, “Delete” target changed to “Back” to give the option to go back to a previous layer in case of an error. For more details of this 4-target speller logic please refer to [20].

#### 2.2.2. Stimulation Frequencies

The list of frequencies displayed was (in Hz): 6.10, 7.15, 8.39, 9.84. The flickering on the monitor screen was generated using the method proposed in [23]. In short: this approach approximates the frequency by utilising two easily realisable (dividers of the monitor vertical refresh rate) neighbouring frequencies and altering between them. The arrangement of the stimuli did not require position randomisation, since there were no differences in the number of neighbouring stimuli. The words to be written by the participants were selected in a way that all three targets containing letters had to be selected equally, six times to spell each word without errors. Additionally, to make the classification more robust, three additional frequencies were considered in the calculations (but these were not shown to the participants) with the frequencies: 6.80, 7.98, and 9.36 Hz. The classification results of these “virtual frequencies” were simply ignored, this resulted in a much more reliable classification of the true stimulation frequencies displayed on the computer screen. For more details on the additional frequencies please refer to [10], where they were originally introduced.

#### 2.2.3. Feedback Methods

For each feedback parameter, minimum and maximum values were determined. The size limits for the stimuli were between 225×225 pixels and 340×340 pixels.

For the contrast, only the stimulus RGB values were modified, the black background remained constant. The stimulus colour values ranged from RGB (128, 128, 128), which corresponds to dark grey, to RGB (255, 255, 255), which is white, and the R, G, and B values always remained equal to each other, i.e., these three RGB values were always changing simultaneously. This resulted in colour changes from dark grey through light grey to completely white according to the probability for that stimulus, as calculated by the classifier.

For the contrast changing, the sizes of the stimuli were kept constant (282×282 pixels). Similarly, for the size changing the contrast was constant, the colour of the stimuli was constantly set to RGB (255, 255, 255), white.

**Figure 3:**
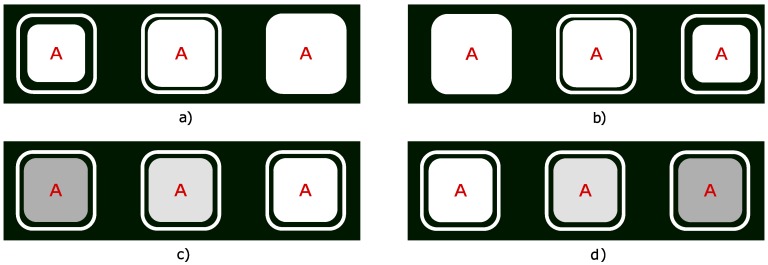
The four used feedback methods: (**a**) “*size increasing*”; (**b**) “*size decreasing*”; (**c**) “*contrast increasing*”; (**d**) “*contrast decreasing*”. The left column depicts how stimuli with a low assigned probability were displayed, the middle column shows stimuli with a probability about half of the threshold, and the right columns shows stimuli with probability at or above the threshold.

This different visual feedback was realised by using the probabilities calculated by the classifiers after each incoming block of EEG data (every 0.5 s). The exact values for the changing parameters were calculated as in our previous study, with the formula:

(1) $$F_{i}=p_{i}/\beta_{i},$$

where $F_{i}$ is the feedback factor, $p_{i}$ is the overall probability of user looking at the stimulus (as calculated by the classifier), and $\beta_{i}$ is the threshold. If the probability was higher than the threshold, $F_{i}$ was set to 1.0. $F_{i}$ was then multiplied by the range of the feedback scale, e.g., in the case of “size” changes 340 pixels − 225 pixels = 115 pixels. If the feedback was “*increasing*”, the product of $F_{i}$ and the range was added to the min. value to get the size/contrast of the stimulus each frame, if the feedback was “*decreasing*”, the calculated value was subtracted from the max. value. Figure 3 explains all four used feedback methods.

### 2.3. Classification

Minimum energy combination method (MEC) [10,24] was used for online SSVEP signal classification. SSVEP power estimations for all of the frequencies were normalized into probabilities. As mentioned above, three additional frequencies were used in the calculations to make the estimations more robust. The MEC method is used to calculate relative probabilities for all frequencies, and if one of the probabilities is higher than the corresponding pre-set threshold, the frequency is classified. Brain signals contain noise, which can influence the probabilities calculated by MEC, even though it is designed to remove a high percentage of the noise. With four frequencies to classify, the thresholds need to be higher, because the chance level is higher, i.e., noise has a greater impact. By adding the three additional frequencies to classify, the effect of noise is partially mitigated. For more details please refer to [10,25]. These additional frequencies were not presented to the users, and could not be selected either.

The classifier did not require training; however, a classification threshold (ranging from 0 to 1.0) was determined individually for each subject before the experiment. For the thresholds (separate threshold for each stimulus), users were instructed to select each target twice, and the thresholds were adjusted until this was achieved without misclassifications and with a reasonable speed. An automated method could be utilised to set up thresholds, such as described in [13]. Since the manual setup required only a few minutes maximum, as only 4 thresholds needed to be set, no automatic method was used in this experiment.

The EEG data were transferred to the PC in blocks of 2500 samples (0.5 s recordings with the sampling rate of 5000 Hz). Down-sampling resulted in blocks of 125 samples (same 0.5 s with 250 Hz sampling rate). The classifications were performed by four separate classifiers. These were of fixed length (2.0 s, 3.0 s, 4.0 s and 5.0 s long, respectively). When enough data were available (more than 5.0 s) all of them were running concurrently classifying after each incoming block of data (every 0.5 s). As an example, after 4.5 s recording the first three classifiers were active, analysing the last 2.0, 3.0 and 4.0 s of data, respectively. The probabilities calculated by the classifiers were combined by weighted averaging, with weights assigned to the classifiers based on the amount of data processed, as more data usually yield more accurate results [25]. For more details please refer to [17].

The probabilities used for overall classification were calculated from the individual classifier results, by calculating the weighted average. If none of the probabilities exceeded the threshold, the output was rejected. If one of the probabilities exceeded the threshold, the following classifier outputs were rejected for a short duration, in order to negate the effect of the previous stimuli on the EEG data, and to allow users to find the next target. The duration of this gaze shifting period was set to 1.5 s. During this period the targets did not flicker, allowing the users to find the desired target and change their focus. As movements cause artefacts in the recorded EEG data, the gaze shifting time also lowers the chance of false classifications. False classifications caused by movements could only occur if the movements lasted long enough, exceeding the allocated gaze shifting time. The length of the gaze shifting time was set based on our previous experience. For more details please see [10].

Moreover, an audio file of the spelled character was played after the third selection step to inform the user what character was selected. The audio file “Select” was following the previous selections or alternatively “Back” and “Delete” when those commands were selected. Compared to visual feedbacks, which were used continuously, audio feedback occurred only after selecting one of the targets (when the classifier output was higher than the threshold). Moreover, the audio feedback was executed during the gaze shifting period; therefore, it only helped users decide on the next target (whether the next letter or “Delete” had to be selected). The users had to correct any mistakes in the written text using the last box, labelled “Delete” or “Back”, which removed the last character of the user-written string or returned to the previous step, respectively.

### 2.4. Experimental Protocol

In this experiment users had to spell altogether five words, each with five different feedback methods: “*no feedback*”, “*size increasing*”, “*size decreasing*”, “*contrast increasing*”, and “*contrast decreasing*”. The order of the spelling tasks was randomised to avoid bias.

**Figure 4:**
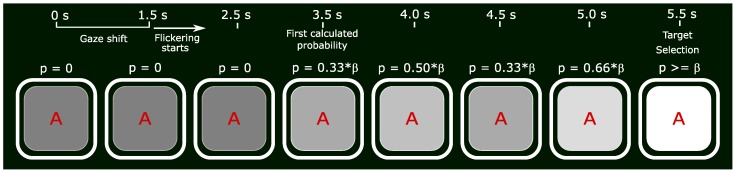
An example time line of target selection using the “*contrast increasing*” scenario, accomplished in 5.5 s. ‘p’ notes the probability calculated by the classifiers, while β notes the corresponding threshold.

As indicated by the name, in the “*no feedback*” condition no information was given to the users regarding classification progress. In the “*size increasing*” and the “*size decreasing*” conditions, the stimuli were getting bigger or smaller, respectively, as the stimulus was assigned a higher probability by the classifier. “*Contrast increasing*” and “*contrast decreasing*” were realised through changing the intensity/shade (RGB values) of the stimulus, which in the off phase was always black, and in the on phase a shade of grey. For a more detailed description please refer to the Section 2.2. Figure 4 shows an example time line of target selection using the “*contrast increasing*” scenario.

After signing the consent forms, users were prepared for the EEG recording. For each user the individual thresholds for classification were set. The values set this way were not altered throughout the rest of the experiment. If no selection was made with a scenario for several minutes, caused by the probabilities calculated by the classifiers not reaching the corresponding threshold, or the classification accuracy was insufficient to continue, the scenario in question was marked as not finished.

The spelling tasks all required six letters, which could be written, in case of no errors, using 3 × 6 = 18 selections. Mistakes had to be corrected. After each spelling task users were asked to rate the used scenario on a Likert scale from 1–10, where 1 noted a very negative impression, and 10 corresponded to a very positive impression. The experiment lasted approximately one hour. This included informing the users about the experiment as well as electrode preparation. Usually these steps took about 20–25 min. The time for the spelling tasks varied greatly, as some scenarios were more difficult to complete (shown by the lower ITRs). The fastest users took a few minutes only to complete all spelling tasks, while in the slowest took 17.15 min. Additionally, users had to rate each scenario and they were allowed to have short breaks between the spelling tasks. As in most cases no breaks were requested, the time between spelling tasks was only 1–3 min long.

The complete word list, from which the five spelling tasks were randomly selected: “COVERS”, “HORSES”, “STRIKE”, “STORES”, “MISTER”, “RUBBER”, “SOLELY”, “LITTLE”, “BUFFER”, “TIMING”, “REFUSE”, “FORCES”, “LIKELY”, “MODIFY”, “FLAVOR”, “LIKING”, “MOSTLY”, “POLICE”, “LOSING”, “CLOSER”, “MERITS”, “OFFICE”, “WIDELY”, “COUPLE”. These were selected according to the criteria described in Section 2.2.

## 3. Results

The achieved accuracies and ITRs are presented in Table 1 and Table 2. The used settings enabled a maximum of 35.12 bit/min ITR (fastest possible selections using minimum classifier length of 2.0 s, 100% accuracy, including gaze shifting times of 1.5 s). For the selection of a single letter, following the previous selection, the minimum time of 10.5 s is necessary (1.5 + 2 + 1.5 + 2 + 1.5 + 2 = 10.5 s). The gaze shifting time was not necessary for the first selection, reducing the time for the first letter by 1.5 s. These result in the maximum spelling speed of approx. 5.7 characters per minute. An online tool for ITR calculation can be found at https://bci-lab.hochschule-rhein-waal.de/en/itr.html.

The mean (SD) ITR was 23.9 (7.1) bit/min, while the mean accuracy was 95.2 (6.3)%. These values show that the BCI was working with high accuracy and reasonably fast most of the time.

Statistical analysis was conducted to investigate performance differences between the different feedback scenarios. By using d’Agostino–Pearson tests for checking the normality of the results we found that ITR results can be used for parametric tests; however, accuracy results require non-parametric equivalents. Therefore, for ITR comparisons repeated measure one-factor ANOVA was used, while for accuracy comparisons Friedman’s test was used. These were followed by pairwise comparisons with paired *t*-tests and Wilcoxon signed ranks test, respectively, when necessary. Sphericity for ITR results was also considered; however, the sphericity assumption was not violated (Mauchly test *p* value was 0.327). Bonferroni correction was used for multiple comparisons.

ITR showed significant difference p=0.002 with the ANOVA. After pairwise comparisons the “*contrast increasing*” scenario proved to be slower than all other scenarios, the “*no feedback*” (*p* = 0.002), the “*size increasing*” (*p* = 0.041), the “*size decreasing*” (*p* = 0.021), and the “*contrast decreasing*” (*p* = 0.002) scenarios. All other pairwise comparisons showed no significant differences. Accuracy, on the other hand showed no significant differences using the Friedman test (*p* = 0.142).

### 3.1. Subject Specific Results

To further investigate our hypothesis, we organized for each subject the ITR from fastest to slowest, and compared the fastest methods to the no feedback condition for that subject. To be able to compare the results statistically, we calculated the ITR value for each step of writing the target word. This means that 18 values were calculated, three for each letter. ITR was used for comparisons as it incorporates accuracy as well as selection times, therefore, it is a practical measure of performance.

To calculate ITR values for each stage, the following methods were used. Firstly, the gaze shifting time was included in all selection times, in order not to generate outliers in the statistical analysis. Regarding accuracy, the same logic was utilised as for the whole words, error corrections were considered correct. ITR values were calculated for each stage. E.g., with the word “COVERS”, an ITR value was calculated upon selecting the target with “ABCDEFGHI” letters, also when selecting “ABC” and when selecting “C” with the 4-target interface. If a letter was accidentally deleted afterward, it had to be rewritten and the next stage reached to calculate the next ITR value.

Paired t-tests were used to compare the ITR values between the fastest and the “*no feedback*” spelling tasks. It is noteworthy, that for some cases the amount of samples is lower than optimal. Therefore, these statistical tests are weaker. This means that there is an increased chance of accepting the null hypothesis, no difference between the feedback methods in our case, while in truth there is a difference. In turn, this means that any differences found significant here would remain significant with more samples. Furthermore, as the highest ITR values are expected to be the same or higher than the “*no feedback*” ITR values and never lower, the one-tail t${}_{crit}$ and *p*-values were used. *p*-values are shown in Table 2, and significant cases are marked with a ‘*’.

Table 3 shows for each feedback method the number of subjects for whom that was the best method. There was a case where the best ITR and the second best ITR did not differ in the first two decimals: S12. As it can be seen from the table the ”*contrast increasing*” scenario is the least often marked as best.

**Figure 5:**
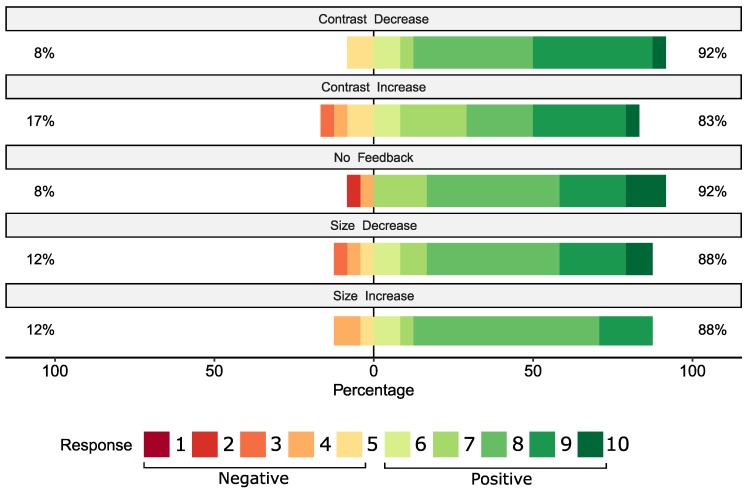
The subjective opinions of all participants. The answers were given on a Likert scale from 1 to 10, where 1 corresponded to a completely negative opinion, while 10 corresponded to a completely positive opinion. Percentage values on the graph note the ratio of scores lower than or equal to 5 for the left side, noting negative opinions, and from 6 to 10 on the right side, representing positive opinions.

The subjective user opinions are shown in Figure 5. The scores were compared with Friedman’s test, p=0.38. Therefore, the subjective opinions do not significantly differ for the feedback modalities.

## 4. Discussion

We investigated the effects of different feedback methods on online BCI performance. This experiment was conducted to examine the effects of different visual feedback with a different number of stimuli. Therefore, we used a 4-target spelling interface with four different feedback scenarios and the “*no feedback*” condition. Our results confirm our hypothesis and the findings of our previous studies, that the effect of user feedback can hugely impact BCI performance. With the used scenarios, the fastest feedback method was found to be significantly faster for 6 users, for them 46.52% faster on average than the “*no feedback*” scenario. Additionally, since differences between ITR values are low, the length of the spelling tasks is not always sufficient, and the statistical analysis became weak, resulting in a high chance of a Type II error. This means possibly more significant differences would have been found with a larger sample size.

We did not find any effect of the visual feedback on classification accuracy, the users were able to complete all tasks with approximately the same amount of mistakes, which confirms the robustness of 4-target spellers. Contrary to our previous studies, with more users even the generalised results showed significant differences, marking the “*contrast increasing*” scenario as significantly slower than most others. That said, there were a few users, for whom this feedback resulted in the fastest selections. This means that even though it is generally slower than other methods, this mechanism should also be considered for further implementations.

As mentioned, the experiments utilising real-time feedback methods in a similar manner are very rare in the literature. There are many occasions when feedback methods are used in another sense, such as in [26], where after each selection the users are provided a visual or auditory representation of the consequences of the selection. In [26] these are representations of robot movements. These types of feedback modalities are similar to the auditory feedback in this paper, in a way that they occur after each target selection and not before.

Feedback methods similar to the ones employed in this paper were used by [18]. They used closed-loop subject-specific selection of the stimulation frequencies together with the closed-loop auditory feedback (the pitch changing with distance from the pre-set threshold) as real-time feedback. This lead to increased BCI ITR performance. The achieved median ITR${}_{max}$ with this system was 17.38 bit/min. In [19], the authors used colour changes of the characters as real-time feedback. They tested together with their new classification method and with dynamic optimisation reached a PITR (SD) value of 41.08 (7.43) bit/min. However, all comparisons should be taken with extreme caution as there are multiple differences between the used systems. Not to mention the different performance metric, PITR, the results of [19] were gotten not solely by feedback methods, but also by using the new classification method. Moreover, in both examples the classification method, and the parameters of classification are different, which creates further difficulties when trying to compare performance metrics. As an example, by setting the gaze shifting time in our paper lower (1.5 s is a really long time and is usually selected for BCI-naive subjects only), all ITR values would increase substantially. As there are multiple different parameters that can influence the end results, the most reliable comparisons can be made within a paper, by looking at the differences between the control condition and the novel methods.

Stimulus parameters can influence the amplitude of the evoked neural responses [6,7,8,14,15] and it is possible that for some of the differences we discovered, the parameters of the stimuli are partially responsible. As an example, for the “*contrast increasing*” scenario, it is possible that the low contrast at the start of the flickering had a negative effect on the performance for some users. Investigating these effects requires an experiment with multiple different constant contrasts and also with changing contrasts (as feedback); however, due to the amount of tests necessary for both size and contrast changes, this would have likely doubled the duration of our experiment, and would have likely introduced bias from fatigue or lack of attention from BCI users. Nevertheless, these effects require further investigation. Furthermore, we suggest that the stimulus parameters such as size and contrast should be also investigated individually for users, as it could also prove to be user dependent whether they improve or degrade BCI performance. If this is true and the optimal feedback methods/stimulus parameters are determined, they could be adapted for each user for reaching an optimal speed with the BCI.

The number of users for whom the “*no feedback*” condition proved to be the fastest, was six. This value is higher than in our previous study, and this could be the consequence of excluding duty-cycle changes (where the feedback was realised by increasing/decreasing the ratio of the on/off phases of the stimuli while keeping the frequency the same), which could have been faster for these users. Moreover, additional mechanisms of feedback could be used, such as changing not the contrast but the colour of the stimuli (e.g., turning from darker to brighter green), or changing the shape of them. Another possibility is that for the 4-target speller the effects of the feedback methods are diminished.

The feedback modalities used in this experiment were chosen according to their simplicity in implementation, as size and colour changes are easily realisable on a computer screen. This makes the ideal for implementation in various SSVEP BCI systems. However, the possible feedback modalities are not limited to these. Changes in shape, colour (not grey-scale), opacity and any other method could be tested as a feedback modality in the future, to hopefully find effective and user-friendly solutions.

Interestingly, although the “*contrast increasing*” scenario was found to be performing significantly worse than the other methods, the user’s subjective scores do not reflect this. This could be a consequence of better user-friendliness or mean that slower performance is not perceptible by the participants. The topic could be further investigated with elaborate questionnaires for more detailed user opinions.

We utilised the same classification method as previously; however, the effect of these feedback modalities with different classification methods is still not investigated, and requires further experiments.

We utilised a 28-target interface with the same feedback methods; however, as explained above, the amount of samples was too short, resulting in a high chance of a Type II error (in statistical hypothesis testing, a type II error is the non-rejection of a false null hypothesis, also known as a “false negative”). This means we missed significant differences because of the low sample count, in the case of this interface only 6 samples. In these results we found for two users significant differences, where the improvement in ITR was 25% and 53.7%, respectively. By employing longer spelling tasks in the future, more robust statistics could be expected, which will likely result in more significant differences discovered. As this would have quite substantially expanded this experiment and would have introduced bias based on user fatigue/user motivation, we refrained from using longer spelling tasks.

## 5. Conclusions

In this experiment we aimed to investigate the effects of feedback methods with 4-target BCI spelling interface, as well as to confirm our previous results, that visual feedback about the classification progress improves online BCI performance of SSVEP-based BCI spellers [17]. We used four feedback methods based on size and contrast changes of the stimuli and compared the best to the control condition (no feedback). Based on our findings, the changes are statistically significant for six users. The average improvement of ITR in these cases was 46.52%. With longer spelling tasks the chances of Type II errors would be lowered, and more significant differences could be discovered.

When the effects of different feedback modalities are averaged for users, the beneficial effects are not noticeable. However, by checking the results individually, the optimal feedback can have a huge impact on ITR. By determining the optimal feedback method for the user, the speed of the BCI can be improved without affecting the accuracy negatively.

This topic needs to be investigated further, most importantly to check whether the optimal method is consistent over time (if the best method is the same after a couple of days or weeks). Methods that are different from those presented here, such as colour or shape changes, could also be investigated, as well as interfaces with more targets.

Our results suggest that by determining the optimal feedback method and using it for the BCI system, ITR can be greatly improved for many users. 

## Figures and Tables

**Table 1:** The accuracy results with the interface. S1–S24 notes the subjects, and the different columns refer to different feedback methods (as compared to the no feedback condition, shown as ‘No FB’).

Subjects	Accuracy [%]				
	No FB	Size Incr.	Size Decr.	Cont. Incr.	Cont. Decr.
**S1**	95.0	100	100	95.5	100
**S2**	100	100	100	100	100
**S3**	95.0	100	95.0	90.9	100
**S4**	100	100	91.7	83.3	95.0
**S5**	90.9	83.3	85.7	70.8	90.9
**S6**	100	100	95.0	90.9	100
**S7**	100	95.5	100	100	95.0
**S8**	90.5	100	91.7	100	86.7
**S9**	100	90.9	87.0	100	100
**S10**	100	88.5	100	100	100
**S11**	100	100	100	100	100
**S12**	95.0	95.0	100	87.5	100
**S13**	100	100	100	95.0	100
**S14**	94.7	87.5	94.7	91.7	91.7
**S15**	100	95.0	100.0	90.9	100
**S16**	100	100	92.3	100	100
**S17**	95.0	95.0	100	100	95.0
**S18**	91.7	95.5	94.7	100	100
**S19**	100	100	91.7	90.9	100
**S20**	100	100	85.2	87.5	91.7
**S21**	100	100	95.0	100	90.9
**S22**	100	100	100	95.0	100
**S23**	78.6	81.5	76.3	84.4	90.9
**S24**	85.7	76.6	95.0	79.4	95.0
**Mean**	**96.3**	**95.2**	**94.6**	**93.1**	**96.8**

**Table 2:** The ITR results with the interface. S1–S24 notes the subjects, and the different columns refer to different feedback methods (as compared to the no feedback condition, shown as ‘No FB’). Lowest and highest ITRs are marked bold for each feedback method. The subject-wise ratio of the fastest method and the “*no feedback*” condition is shown with the ITR results. *p*-values show the significance of the subject-wise comparisons, described in Section 3.1. Significant differences are marked with an ‘*’.

Subjects	ITR [bit/min]						
	No FB	Size Incr.	Size Decr.	Cont. Incr.	Cont. Decr.	*p*-Value	Fastest/No FB [%]
**S1**	26.5	29.0	30.0	20.5	30.7	0.411	115.8
**S2**	31.1	**33.3**	34.4	**32.5**	32.3	0.038	110.6 *
**S3**	22.7	28.7	24.6	18.5	27.9	0.181	126.4
**S4**	32.1	27.7	22.5	13.0	26.0	-	100.0
**S5**	19.1	12.0	11.1	**5.1**	15.6	-	100.0
**S6**	32.5	31.4	26.7	20.0	31.8	-	100.0
**S7**	25.0	22.5	30.9	29.4	23.8	0.003	123.6 *
**S8**	22.3	27.9	12.8	23.9	16.4	0.441	125.2
**S9**	27.7	13.2	16.1	24.4	27.9	0.452	100.7
**S10**	31.8	19.0	30.3	28.7	32.3	0.445	101.6
**S11**	**33.6**	28.7	32.5	25.9	32.1	-	100.0
**S12**	25.9	24.9	34.1	19.1	**34.1**	0.015	131.7 *
**S13**	28.3	24.7	**34.4**	20.5	32.8	0.001	121.6 *
**S14**	15.6	14.9	19.8	11.2	**14.1**	-	100.0
**S15**	32.1	23.9	30.3	17.7	29.6	-	100.0
**S16**	27.4	26.4	15.5	27.7	25.2	0.272	101.1
**S17**	23.8	25.0	31.1	27.8	23.2	0.153	130.7
**S18**	16.0	20.0	21.4	24.7	23.4	0.097	154.4
**S19**	28.5	30.3	23.0	15.5	29.5	0.090	106.3
**S20**	22.1	23.3	13.2	13.3	16.0	0.471	105.4
**S21**	27.4	26.7	26.4	26.4	19.7	-	100.0
**S22**	33.3	29.2	29.0	22.6	30.7	-	100.0
**S23**	**7.2**	**10.7**	**7.4**	11.1	17.4	0.001	241.0 *
**S24**	16.6	12.4	24.7	12.0	25.0	0.035	150.6 *
**Mean**	**25.4**	**23.6**	**24.3**	**20.5**	**25.7**	-	-

**Table 3:** The number of subjects for whom the specific feedback method resulted in the highest ITR.

	Cont. Decr.	Cont. Incr.	Size Decr.	Size Incr.	No FB
Fastest modality	6	2	6	4	6
